# Investigation of Free-Standing Plasmonic Mesoporous Ag/CMK-8-Nafion Composite Membrane for the Removal of Organic Pollutants with 254-nm UV Irradiation

**DOI:** 10.1186/s11671-017-2124-7

**Published:** 2017-05-19

**Authors:** Chuan Ming Tseng, Hsin Liang Chen, Sz Nian Lai, Ming Shiung Chen, Chien Jung Peng, Chia Jui Li, Wei Hsuan Hung

**Affiliations:** 1 Department of Materials Science and Engineering, Feng Chia University, Taichung, 407 Taiwan; 2 Department of Materials Engineering, Ming Chi University of Technology, New Taipei City, Taiwan

## Abstract

“Carbon-based material” has demonstrated a great potential on water purification due to its strong physical adsorption to organic pollutants in the water. Three-dimensional cubic ordered mesoporous carbon (CMK-8), one of the well-known ordered mesoporous carbons, was prepared by using nanocasting method with mesoporous silica (KIT-6) as the template. In this study, CMK-8 blended with Nafion polymer to form a free-standing mesoporous CMK-8-Nafion composite membrane. The synthesis of high crystallinity CMK-8 was characterized by X-ray diffraction (XRD) and transmission electron microscopy (TEM). More than 80% methyl orange (MO) removal efficiency was observed under 254-nm UV irradiation after 120 min. Ninety-two percent recycling performance was remained after four recycling tests, which indicated a reliable servicing lifetime for the water purification. Furthermore, an additional layer of plasmonic silver nanoparticles (Ag NPs) was integrated into this CMK-8-Nafion membrane for higher pollutant removal efficiency, attributing from the generation of plasmon-resonance hot electrons from Ag NPs. A 4-in. CMK-8-Nafion composite membrane was also fabricated for the demonstration of potential large-scale utilization.

## Background

**Scheme 1:**
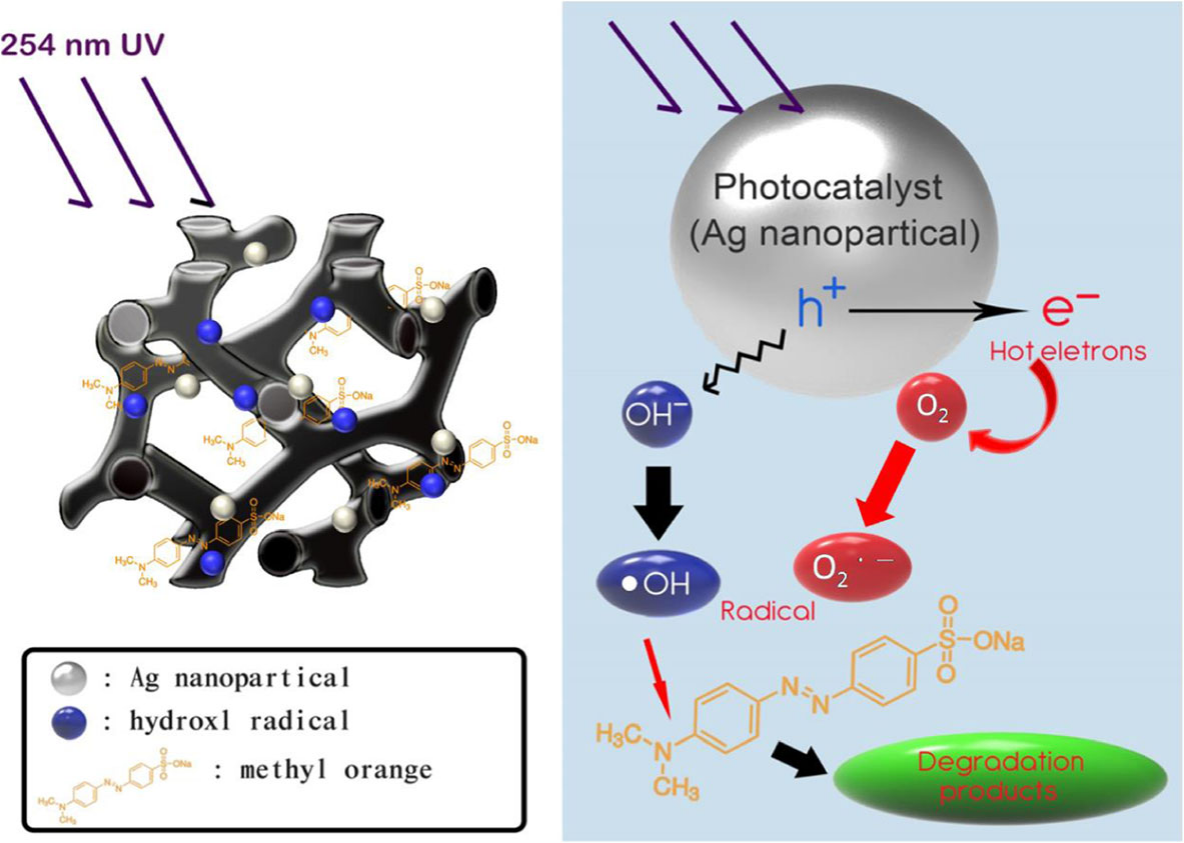
Enhanced MO decomposition mechanism and reaction route in an Ag/CMK-8-Nafion system

Maintaining a constant supply of clean water has become a vital issue in this decade because of the increasing number of contaminants produced from industrial wastes worldwide. Various organic contaminants such as dioxin, ethylbenzene, and polycyclic aromatic hydrocarbons are frequently found in wastewater and are substantially harmful to human health and ecological security [1, 2]. Therefore, development of efficient methods for decontamination and disinfection of water, particularly of drinking water sources, is urgently required. Several approaches have been adopted for removing organic pollutants from water. For example, reverse osmosis [3], ion exchange process [4], biochemical processes [5], and physical adsorption [6, 7] are generally used for water purification. Of these technologies, physical adsorption is the most commonly used because of its low cost and easy operation. Carbon-based materials [8, 9] such as activated carbon and carbon nanotubes are promising candidates for water purification because of their exceptional capabilities of adsorbing various organic contaminants through numerous bonding types, such as electrostatic interactions, π–π bonding, hydrogen bonding, and hydrophobic interactions [10, 11]. Organic water pollutants can also be photocatalytically decomposed with semiconductor materials that exploit the electron-hole pairs (excitons) from the conversion of incident photons. These high-energy electrons and holes react with aqueous solutions at solid and solution interfaces to generate •OH and O_2_^•−^, triggering the decomposition of organic pollutants in wastewater [12]. Scheme 1 illustrates a possible explanation for the enhanced mechanism and reaction route in an Ag/CMK-8-Nafion system. Under photon irradiation at 254 nm, hot electrons and holes are excited from the surfaces of silver nanoparticles (NPs) to produce superoxide radical anions (O_2_^•−^) and hydroxyl radicals (•OH), respectively; the primary oxidizing species correspond to photocatalytic oxidation processes [13–15].

In addition to activated carbon and carbon nanotubes, ordered mesoporous carbon is another effective material used for removing pollutants from wastewater [16–19]. Ordered mesoporous carbon has been receiving much attention because of its high surface area, high conductivity, and highly uniform and regular pore sizes, which facilitate mass transport [20–23]. Moreover, ordered mesoporous carbon has been successfully employed in energy storage devices such as fuel cells [24–26] and supercapacitors [27]. Different structures, sizes, and shapes of ordered mesoporous carbon can be explicitly synthesized by varying fabrication parameters and surfactant concentrations [28, 29]. In this study, we propose the application of a free-standing CMK-8-Nafion composite membrane in the photo-induced decomposition of methyl orange (MO). Mesoporous carbon CMK-8 can not only adsorb MO [30] but can also effectively absorb photons due to their blackbody property [31, 32], contributing to the additional photo-induced decomposition of MO [16]. This unique dual mechanism consisting of physical adsorption and photocatalytic decomposition is discussed under different experimental conditions. Finally, we also layered silver plasmonic NPs [33, 34] onto this free-standing mesoporous CMK-8-Nafion composite membrane to further enhance the removal of organic water pollutants.

## Methods

High-quality samples of mesoporous silica KIT-6 and the corresponding mesoporous carbon CMK-8 were prepared using a process similar to that used in previous studies [35]. CMK-8 has a reversed cubic structure, which was then replicated using KIT-6 as a hard template. A dilute H_2_SO_4(aq)_ solution was added to sucrose solution with weight ratios of 1 g KIT-6/1.25 g sucrose/5 g H_2_O/0.14 g H_2_SO_4_. The colloid mixture was dried at 333 K for 6 h and dehydrated at 433 K for 6 h. The aforementioned steps were repeated again with a mixture of 0.8 g sucrose/3.2 g H_2_O/0.09 g H_2_SO_4_. The resultant dark brown powders were carbonized under argon atmosphere at 1173 K for 1 h. The silica template was removed with 1 M hydrofluoric acid in a solution of 50% ethanol and 50% H_2_O, and CMK-8 was finally collected. For the fabrication of CMK-8-Nafion composite membranes, designated amounts of CMK-8 were mixed with Nafion solution at a solid-content ratio of 30%, and this CMK-8-Nafion precursor was ultrasonically agitated for 10 min before the casting step. Each mesoporous CMK-8-Nafion membrane was formed by pouring CMK-8-Nafion precursor solution in a 4-in. petri dish and was then solidified at 323 K for 40 min. In addition, for the deposition of the silver NP layer, a precursor composed of silver acetylacetonate weighing 0.035 g [Ag(acac); 98%, Acros] was dissolved in 30 mL deionized water mixed with 5 mL 99.5% alcohol and the prepared mesoporous CMK-8-Nafion membrane was then immersed in the solution for 15 h [36]. After examination of several samples, the average thickness of the Ag/CMK-8-Nafion membrane was 0.3–0.4 mm. The microstructures and morphologies of KIT-6 and CMK-8 were examined using a scanning electron microscope (SEM; FE-SEM; HITACHI S-4800) and a transmission electron microscope (JEOL JEM-2100F). The pore sizes and specific surface areas were analyzed using N_2_ adsorption/desorption analysis under 77 K (Micromeritics; ASAP2020). The mesostructures of KIT-6 and CMK-8 were confirmed by small-angle (2*θ* of 0.5°–8°) powdered X-ray diffraction (XRD) by using Cu Kα radiation (*λ* = 0.154 nm; scan rate of 1°/min). The chemical states of silver NPs were examined using an X-ray photoelectron spectroscope (XPS; ULVAC-PHI Versa-probe) with Al Kα X-rays and a 45° photoelectron takeoff angle. A 1-eV flooding electron source and 7-eV Ar^+^ was applied for charge compensation during spectrum acquisition. Finally, a UV-Vis integrating sphere was used to evaluate the performance of organic pollutant decomposition by the free-standing mesoporous CMK-8-Nafion membranes under UV irradiation at 254 nm.

## Results and Discussion

**Fig. 1:**
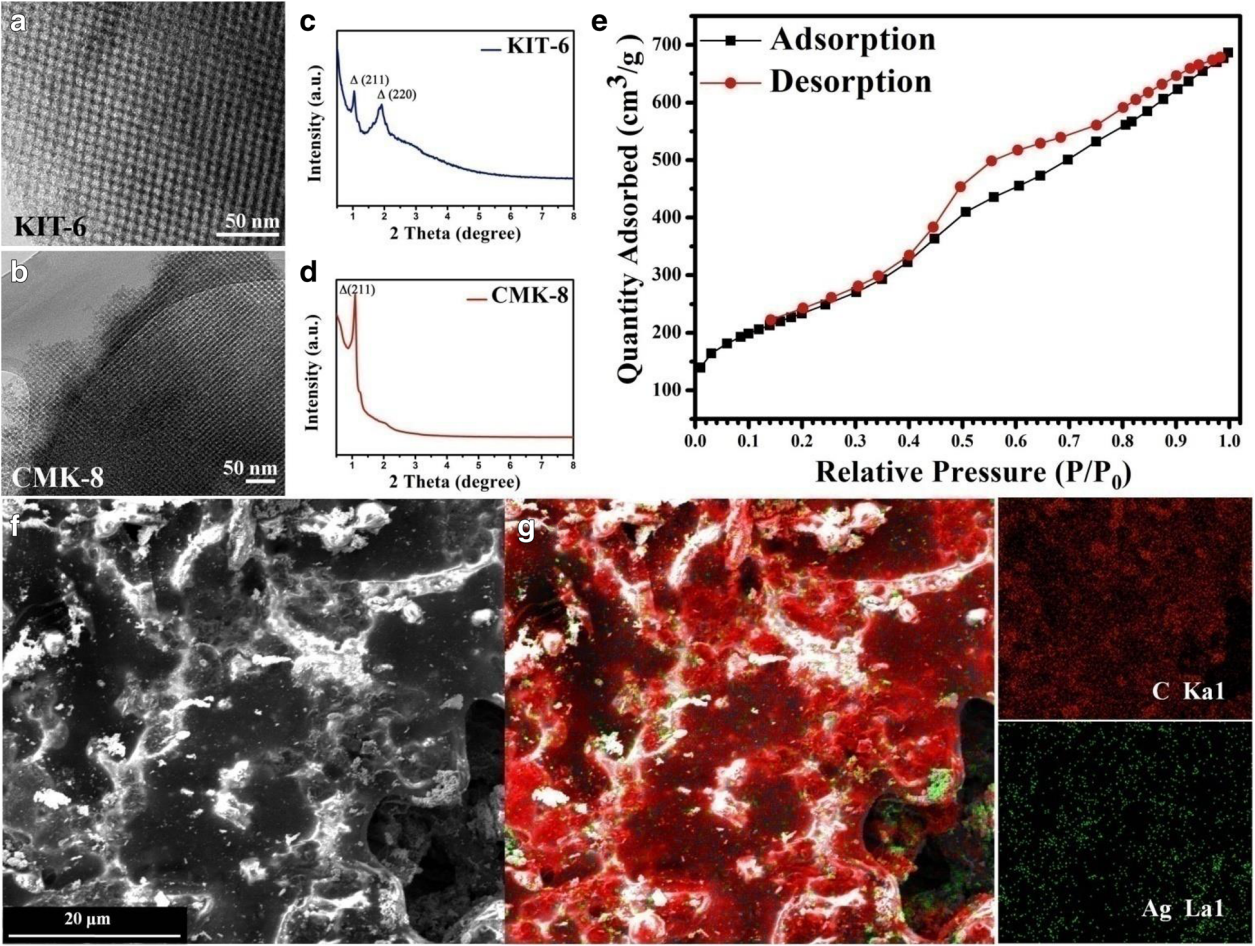
Characterization of KIT-6 and corresponding CMK-8. **a**, **b** High-magnification TEM images. **c**, **d** Small-angle XRD patterns. **e** N_2_ adsorption/desorption isotherms (77 K) of CMK-8. **f**, **g** SEM image and corresponding EDX elemental mappings of C, Ag, and Ag/CMK-8-Nafion membranes

Figure 1a, b presents high-resolution TEM images of mesoporous microstructures of silica KIT-6, and the corresponding three-dimensional (3D) structures of ordered mesoporous carbon CMK-8 exhibited a well-ordered honeycomb structure with a uniform pore size. As depicted in Fig. 1c, d, long-range ordering porosity of KIT-6 and CMK-8 can be observed from XRD patterns in the low-angle range; this finding is in agreement with the result of HRTEM. Because of the high surface area of 3D cubic CMK-8, fast mass transfer kinetics become possible, which is not ably beneficial for organic molecule adsorption. The N_2_ adsorption/desorption isotherms (77 K) were measured for examining the specific surface area of CMK-8. The N_2_ adsorption/desorption isotherms (77 K) of CMK-8 exhibited an essentially type-IV isotherm (according to the IUPAC classification) with a broad hysteresis loop, which had the typical characteristics of capillary condensation in mesoporous channels (Fig. 1e). In addition, according to the Brunauer–Emmett–Teller method, CMK-8 possessed a specific surface area of 840.67 m^2^ g^−1^. The Barrett–Joyner–Halenda analysis of the desorption branch of the isotherm indicated that the pores had an average diameter of approximately 4 nm. CMK-8 is expected to provide a substantial number of active sites for physical adsorption of organic pollutants because of its high specific surface area and porous nature. To fabricate an Ag/CMK-8-Nafion membrane, these CMK-8 powders were mixed with Nafion solution, followed by a chemical reduction process for the deposition of Ag NPs. The morphology of the Ag/CMK-8-Nafion membrane was examined using an SEM, and the results are presented in Fig. 1f. The corresponding energy-dispersive X-ray spectroscopy mapping of C and Ag elements was also performed; the Ag NP distributions on the surfaces of CMK-8-Nafion membranes were clearly observable (Fig. 1g).

**Fig. 2:**
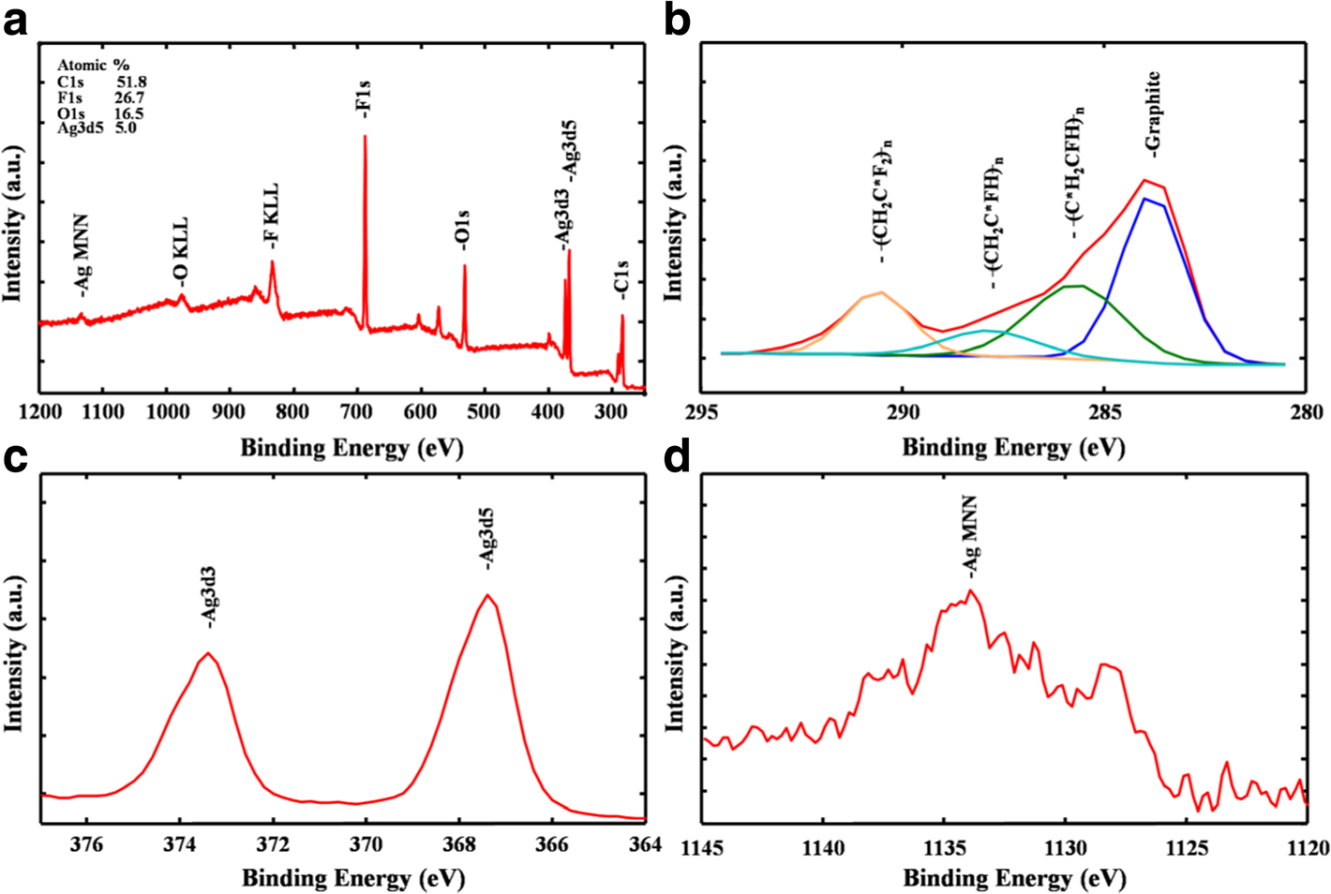
X-ray photoelectron spectroscopy spectra of the Ag/CMK-8 membrane. **a** Survey spectrum. **b** C1s. **c** Ag3d. **d** Ag MNN

To obtain substantial hot electron generation from a plasmonic resonance process, it is essential to preserve the neutrality of silver NPs because neutral silver NPs provide a more severe plasmon resonant condition than oxidized silver NPs do; therefore, characterizing the surface chemical state of silver NPs is crucial. XPS spectroscopy was used for examining the Ag/CMK-8-Nafion system. The elemental survey spectrum of the Ag/CMK-8-Nafion membrane was measured, and the four main elements, namely Ag, C, F, and O, were identified (Fig. 2a). On the basis of this typical survey, the atomic percentage of Ag was estimated to be approximately 5.0%. The high-resolution spectrum of C1s was obtained and is presented in Fig. 2b. The nonsymmetrical peak shape indicated that multiple chemical states of carbon were present in the Ag/CMK-8-Nafion sample; hence, deconvolution was performed to identify each component. The most pronounced peak, located at 284.3 eV, was attributed to the C–C graphitic bonding of CMK-8, indicating that no chemical reactions occurred between silver NPs and CMK-8. The peak at 285.9 eV can be assigned to the carbon bonded to C^*^H_2_CFH_n_, and the peak at 288.0 eV can be attributed to CH_2_C^*^FH_n_. Another broad peak at 290.8 eV can be considered as the superposition of signals from –CF_2_–, –OCF–, and –OCF_2_– groups. As presented in Fig. 2c, a symmetrical Ag3d5 peak was obtained in the high-resolution scan, indicating that Ag NPs were successfully reduced on the surface of the CMK-8-Nafion membrane through a physical adsorption approach without any other chemical bonding. Furthermore, because of the binding energy of Ag, Ag oxides and Ag fluorides differed by only a few tenths of an eV. Thus, determining the oxidation of Ag only by using the Ag3d5 peak position is difficult. For more accurate characterization, Ag MNN auger electrons were also examined. A cross-comparison of simultaneous measurements of the Ag3d binding energy and Ag MNN kinetic energy (KE) peak can determine the chemical state and prevent the confusion of shifts. The Ag MNNKE is given as follows:

1 $$ \mathrm{K}\mathrm{E}\left(\mathrm{eV}\right)=\mathrm{Photon}\ \mathrm{E}\mathrm{nergy}-\mathrm{Binding}\ \mathrm{E}\mathrm{nergy} $$

According to the Al Kα X-ray photon energy (1486.6 eV), the AgM_5_N_5_N_5_ binding energy was calculated as 1133.8 eV. As presented in Fig. 2d, the KE of AgMNN was 358.8 eV (6.0 eV added to the KE data on M_5_N_5_N_5_ to obtain the KE of M_4_N_5_N_5_) [37, 38]. The auger interpretation of the binding energy (367.39 eV; Fig. 2c) and the KE of AgMNN (358.8 eV) indicate the existence of metallic silver NPs in our sample, suggesting that the sample has active plasmon resonance and substantial hot electron generation.

**Fig. 3:**
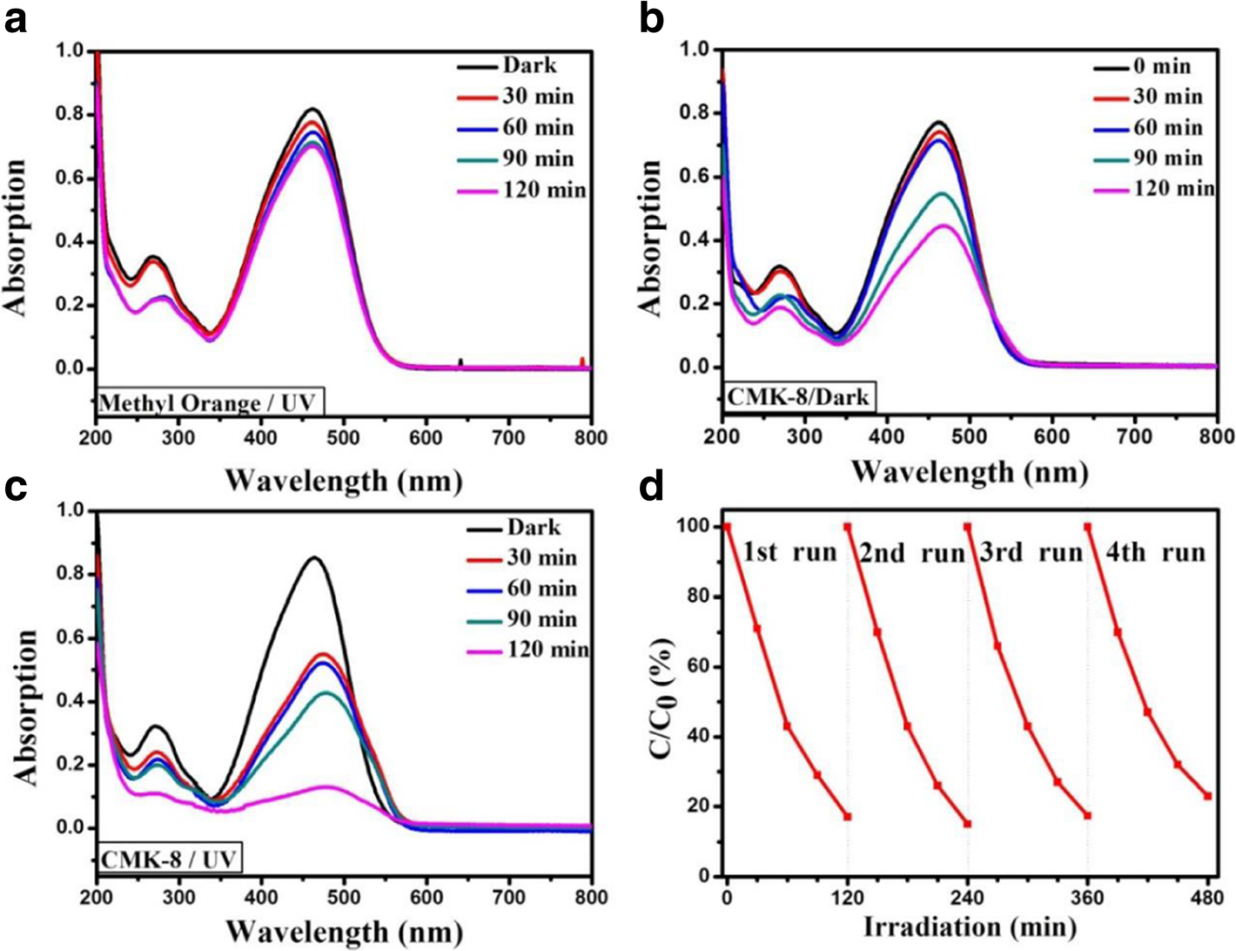
UV-Vis spectra evolution of MO decomposition **a** under UV irradiation at 254 nm only, **b** with the CMK-8-Nafion membrane in darkness, **c** CMK-8-Nafion membrane under UV irradiation at 254 nm, and **d** recycling examination

To investigate the efficiency and mechanism of MO decomposition, we performed three experimental setups for testing the removal rates of MO: (i) UV irradiation at 254 nm with no membrane, (ii) a CMK-8-Nafion membrane only in darkness, and (iii) a CMK-8-Nafion under UV irradiation at 254 nm. Figure 2a–c illustrates the evolution of the UV-Vis spectra of the MO solution under these three decomposition conditions. The absorption peak of MO at 463 nm was obtained from the conjugated structure constructed through an azo bond. A decrease in the peak intensity indicated the decomposition of MO and decoloration of the solution. MO demonstrated a very slight self-degradation under UV irradiation at 254 nm after 120 min (Fig. 3a). When the CMK-8-Nafion membrane was examined in darkness, it exhibited a strong physical adsorption ability for MO even without UV irradiation. A noticeable decrease at an absorption peak of 463 nm was observed with the passage of time (Fig. 3b). Photo-induced decomposition of MO was examined by placing a CMK-8-Nafion membrane under irradiation at 254 nm (Fig. 3c). The MO decomposition efficiency of this photo-enhanced process was nine times higher than that of CMK-8 in darkness, suggesting that the photo-induced decomposition process was achieved by incident photons with preadsorbed MO molecules on the CMK-8-Nafion surface. In addition, we examined the recycling stability of the CMK-8-Nafion membrane (Fig. 3d). The results demonstrated that the CMK-8-Nafion membrane still retained 92% of its original efficiency after four consecutive 120-min decomposition cycles.

**Fig. 4:**
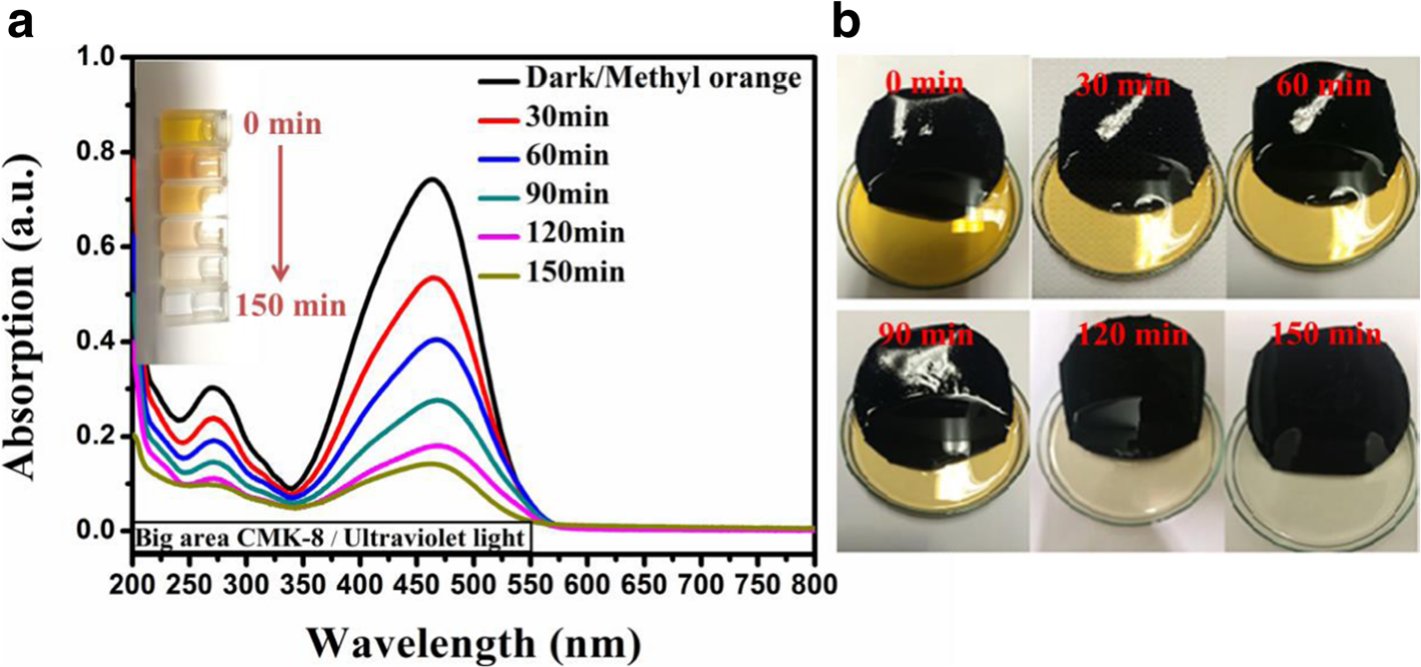
A free-standing 4-in. CMK-8-Nafion membrane. **a** UV-Vis spectrum evolution of MO decomposition. **b** Photographs of MO decoloration under UV irradiation at 254 nm

We fabricated a 4-in. free-standing CMK-8-Nafion membrane to demonstrate the potential practical use of these mechanisms. A similar evaluation process was performed on this 4-in. CMK-8-Nafion membrane. Figure 4a presents the UV-Vis spectrum evolution of MO decomposition under UV irradiation at 254 nm. After 150 min of irradiation, more than 80% of organic MO was successfully removed from the solution. Figure 4b depicts the corresponding photographs of the decoloration of the MO solution with an increase in irradiation time. The 4-in. CMK-8-Nafion membrane had a robust framework of CMK-8 and Nafion, which did not leave any unnecessary legacy products in the cleaned water even after several recycling tests, eliminating the additional effort of removing photocatalytic filter detritus.

**Fig. 5:**
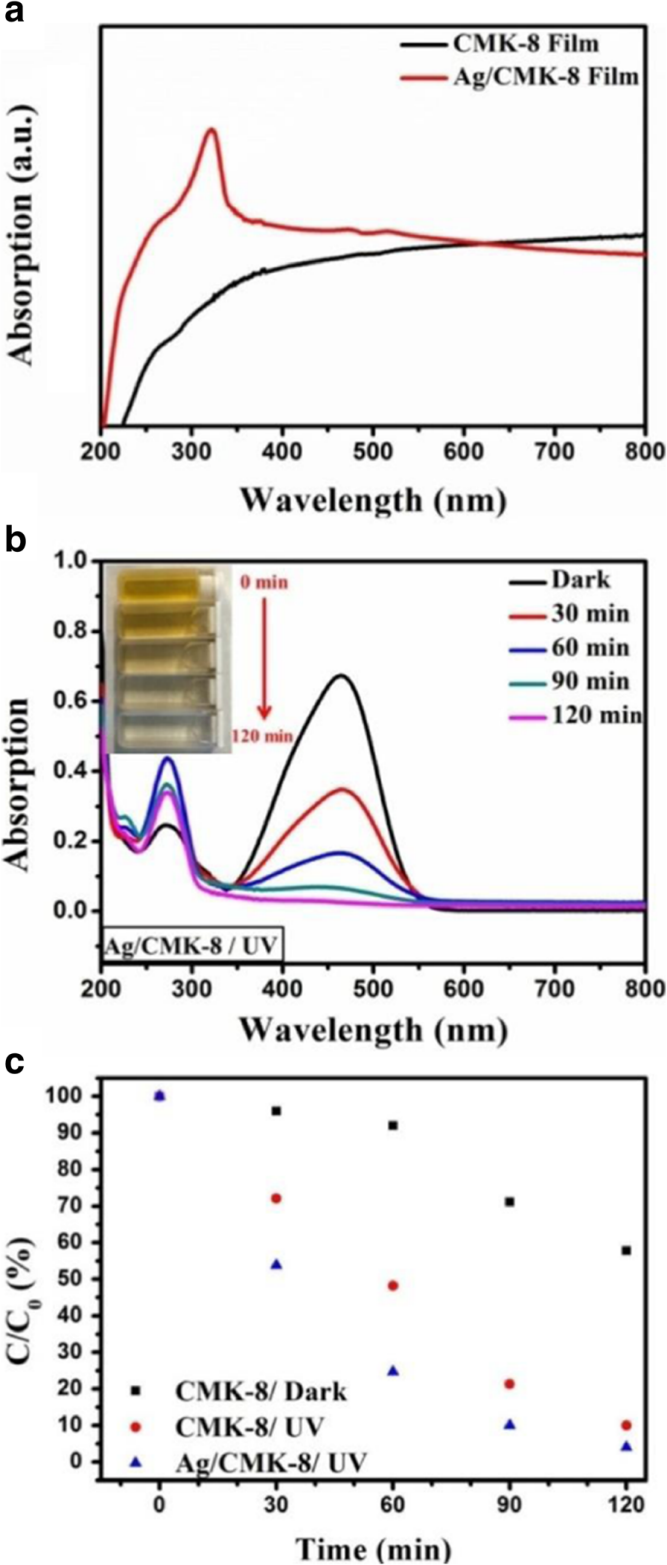
**a** Absorption spectra of CMK-8-Nafion with and without silver NPs. **b** UV-Vis spectrum evolution of MO decomposition with the Ag/CMK-8-Nafion membrane. **c** A comparison of MO decomposition efficiency levels with different experimental conditions

To further improve the MO decomposition process, we introduced a layer of plasmonic silver NPs onto the surface of the CMK-8-Nafion membrane to achieve additional MO decomposition efficiency by the generation of hot electrons from plasmon-resonance NPs. Silver NPs were prepared using a previously reported chemical reduction process. Figure 5a presents the absorption spectrum of CMK-8-Nafion and Ag/CMK-8-Nafion membranes. The CMK-8-Nafion membrane exhibited a typical broad photon absorption from 350 to 800 nm because of the blackbody characteristics of CMK-8. In the Ag/CMK-8-Nafion membrane sample, an additional pronounced silver plasmonic absorption peak [39] was observed at approximately 310 nm, presenting a slight blueshift caused by the low dielectric constant of CMK-8. With the integration of silver NPs, more than 98% MO decomposition was achieved within 120 min under UV irradiation at 254 nm (Fig. 5b). This decomposition enhancement of approximately 20% is attributable to hot electrons generated on the surfaces of silver NPs by the plasmon decay process, substantially raising the population of active radicals in the solution and providing an additional reaction route for the MO decomposition process. Notably, because the excellent molecule adsorption ability of the CMK-8 also provides a perfect reaction ground for these active oxidizing species with preadsorbed MO molecules, the whole decomposition reaction proceeds with the positive feedback of an avalanche. Finally, we compared the decomposition efficiency of CMK-8-Nafion in darkness and under UV irradiation and the efficiency of Ag/CMK-8-Nafion under UV irradiation (Fig. 5c). As expected, the Ag/CMK-8-Nafion sample exhibited the highest MO decomposition efficiency because of additional hot electrons and holes generated from silver NPs.

## Conclusions

Free-standing CMK-8-Nafion membranes were fabricated for improving MO decomposition in wastewater. The basic membrane removed pollutants with an efficiency level of more than 80% after 120 min of UV irradiation at 254 nm. A reliability test indicated that the basic CMK-8-Nafion membrane still retained 92% of its original efficiency after four consecutive MO decomposition processes. Furthermore, with the integration of a silver NP layer, 98% MO decomposition efficiency was achieved, which was approximately 20% higher than that of the basic CMK-8-Nafion membrane. Finally, we demonstrated the feasibility of fabricating a 4-in. free-standing CMK-8-Nafion membrane for high-throughput wastewater treatment.
